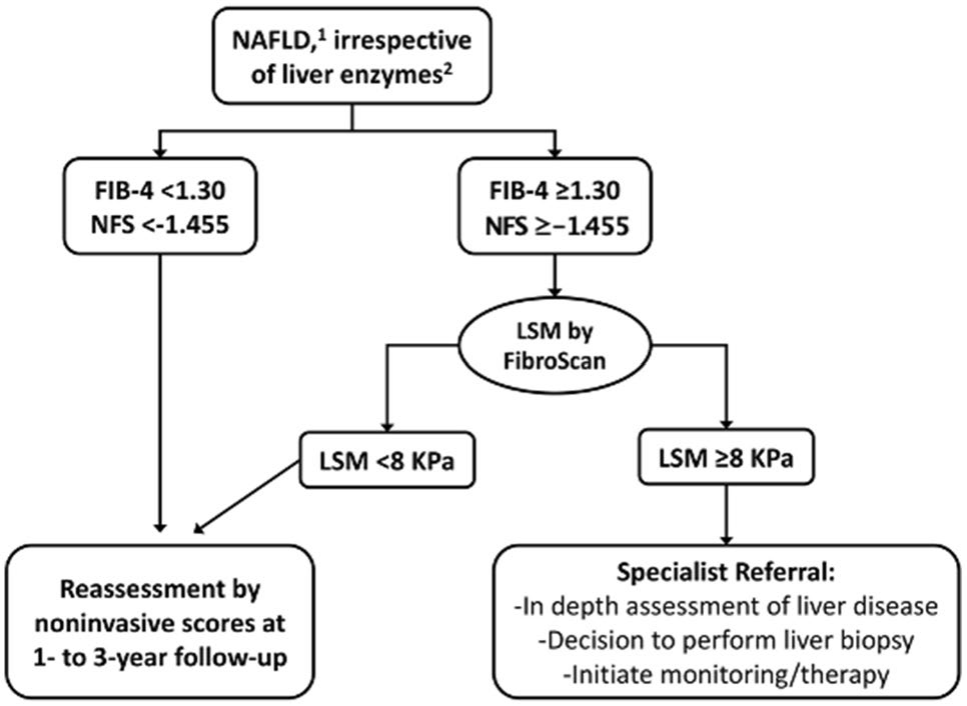# Correction: Non-alcoholic fatty liver disease in adults 2021: A clinical practice guideline of the Italian Association for the Study of the Liver (AISF), the Italian Society of Diabetology (SID) and the Italian Society of Obesity (SIO)

**DOI:** 10.1007/s40519-023-01543-6

**Published:** 2023-03-02

**Authors:** Associazione Italiana per lo Studio del Fegato (AISF), Società Italiana di Diabetologia (SID) and Società Italiana dell’Obesità (SIO)

**Affiliations:** University of Palermo, Palermo, Italy

**Correction: Eating and Weight Disorders - Studies on Anorexia, Bulimia and Obesity (2022) 27:1603–1619** 10.1007/s40519-021-01287-1

In this article, Figure 1 contains an error. The correct rule-in cut-off of NAFLD Fibrosis Score indicating patients at intermediate-high risk of advanced fibrosis should read ≥  − 1.455. The correct figure is reported below. The authors apologise for the mistake.